# Filaggrin gene mutation associations with peanut allergy persist despite variations in peanut allergy diagnostic criteria or asthma status

**DOI:** 10.1016/j.jaci.2013.03.043

**Published:** 2013-07

**Authors:** Yuka Asai, Celia Greenwood, Peter R. Hull, Reza Alizadehfar, Moshe Ben-Shoshan, Sara J. Brown, Linda Campbell, Deborah L. Michel, Johanne Bussières, François Rousseau, T. Mary Fujiwara, Kenneth Morgan, Alan D. Irvine, W.H. Irwin McLean, Ann Clarke

**Affiliations:** a Division of Dermatology, Department of Medicine, McGill University, Montreal, Quebec, Canada; b Division of Clinical Epidemiology, Department of Medicine, McGill University, Montreal, Quebec, Canada; c Lady Davis Institute for Medical Research, Jewish General Hospital, Montreal, Quebec, Canada; d Department of Oncology and the Department of Epidemiology, Biostatistics and Occupational Health, McGill University, Montreal, Quebec, Canada; e Division of Dermatology, University of Saskatchewan, Saskatoon, Saskatchewan, Canada; f Division of Allergy and Clinical Immunology, Department of Pediatrics, McGill University, Montreal, Quebec, Canada; g University of Dundee, Dundee, United Kingdom; h Laval University, Quebec City, Quebec, Canada; i Departments of Human Genetics and Medicine, McGill University, Montreal, Quebec, Canada; j Research Institute of the McGill University Health Centre, McGill University, Montreal, Quebec, Canada; k Trinity College Dublin, Dublin, Ireland; l Division of Allergy/Clinical Immunology, Department of Medicine, McGill University, Montreal, Quebec, Canada

To the Editor:

Recently, our research team found a strong and significant association between loss-of-function (LOF) mutations in filaggrin (*FLG*), a gene that encodes a skin barrier protein, in European and Canadian individuals with peanut allergy (PA).^1^ These mutations result in a barrier defect and have been associated with atopic dermatitis, asthma, and allergic rhinitis.^2^ This finding represents the strongest genetic risk factor found to date for PA, a highly heritable disease,^3^ with an estimated odds ratio (OR) between 1.9 (Canadian) and 5.3 (English, Dutch, and Irish combined).^1^ Because there is no uniformly accepted definition of PA short of oral food challenge, it is worthwhile to examine whether the association between the *FLG* LOF mutations and PA varies with the diagnostic criteria for PA. Furthermore, the frequent coexistence of PA with other atopic conditions may mean that the association between PA and *FLG* LOF mutations is confounded. Although we controlled for eczema in the European populations in our previous work, data on eczema were not available for the Canadian control group; however, data on asthma were available. Asthma is a potential confounder, as it has known relationships with both PA^4^ and *FLG* mutations.^5^ By using statistical sensitivity analyses, we examined the effect of PA diagnostic criteria and asthma on the relationship between PA and *FLG* LOF mutations in a Canadian PA case group. Because the PA case group was composed of both English- and French-speaking individuals, we also investigated whether the difference in OR between the Canadian and European populations could be due to some common French-Canadian mutations not yet identified in *FLG*.

Caucasian subjects from a well-described Canadian pediatric PA case group were recruited (n = 679), and DNA was isolated from salivary samples.^1^ One control group consisted of adult Caucasians recruited from the general population of Ontario, Canada; DNA was provided by the Ontario Population Genomics Platform at The Centre for Applied Genomics (Toronto) (n = 894). A second control group of newborn babies from Quebec City was sampled on the basis of French-Canadian surname (stored blood; n = 268).^6^ All samples were genotyped in Dundee, Scotland, for the 4 most common *FLG* LOF mutations found in Caucasians (R501X, 2282del4, R2447X, and S3247X). rs and accession numbers for mutations are available in the Online Repository at www.jacionline.org (see Table E1).

“Mutation carriers” were defined as those with heterozygous, homozygous, or compound heterozygous mutations. Those individuals with none of the 4 *FLG* mutations were classified as “nonmutation carriers.” The association between mutation status and PA was compared with the Ontario control group, the Quebec control group, and the combined control group. To evaluate whether the association between PA and mutation status changed with case definition, a continuum of PA case definitions was constructed and the resulting OR trends with case definition were examined by using the combined control groups. The methodology and rationale for these definitions are given in the Online Repository available at www.jacionline.org (see Tables E2 and E3). To increase power, case definitions were transformed into an ordered variable and the association between PA and *FLG* mutations was examined by using regression modeling through increasingly stringent definitions of PA to see whether the OR changed significantly.

We also examined the effect of asthma on *FLG* mutation status and PA. Logistic regression using the Ontario control group was conducted with PA status, age, sex, asthma, and interaction terms for PA and age, PA and sex, and PA and asthma. Because there may be interaction between mutation status, asthma, and smoking,^7^ and self-identified asthmatic patients may have significant misclassification, analyses controlled for smoking history in the controls and misclassification in the asthma variable in both controls and cases. A constructed atopic asthma variable was used to control for the effect of smoking with 4 assumptions: (1) Those individuals who have atopic asthma in childhood are less likely to smoke as adults. (2) Those adults who have asthma and have never smoked are more likely to have atopic asthma. (3) If a patient reports bronchial emphysema, he or she does not have atopic asthma. (4) Asthma reported in the case group is atopic. To examine the possibility of error due to self-report of asthma, we completed a sensitivity analysis taking into account data from the PA registry that found that 6% had “forgotten” previously noted atopic history, while 12% more reported atopic history on the current questionnaire than at baseline registry recruitment. Random sampling and subsequent logistic regression modeling was completed 100 times to see the overall effect of error in asthma reporting on the relationship of PA and *FLG* LOF mutations using these parameters.

Finally, a similar sensitivity analysis of PA status was conducted, as PA status could be affected by the age disparity between the Ontario control group and the cases, because it is estimated that up to 20% of individuals with PA may have resolution of their allergy.^8^ This sensitivity analysis also took into account the 1% prevalence of PA in the general population.^9^

Of the 679 cases eligible to participate, 99.3% had good quality DNA. Demographic information is presented in Table I. The 674 cases had approximately twice as many mutations as the controls (20% vs 11%), and the OR for PA and *FLG* mutation status was similar in the Ontario and Quebec control groups (Table II). The genotype frequencies in cases and controls are shown in the Online Repository available at www.jacionline.org (see Table E4). Among the 13 case definitions, there was no significant difference in the OR for the relationship between PA and *FLG* mutation status. Seven representative case definitions are shown in Table III. Logistic regression of the ordered case definition criteria variable produced similar results.

**Table I:** Demographics: PA cases and controls

Characteristic	PA cases	Ontario controls	Quebec controls
No. of subjects	674	894	268
Age (y)∗			
Mean ± SD	9.3 ± 4.0	65.5 ± 10.2	9.7 ± 0.5
Range	0-21	33-84	7-10
No. with missing data	0	2	0
Sex			
No. of males	416	281	157
Proportion of males (CI)	0.617 (0.580-0.654)	0.315 (0.284-0.346)	0.586 (0.526-0.645)

∗ Age on January 1, 2009.

**Table II:** *FLG* genotypes and statistical tests of PA cases compared with control groups

	PA cases	Ontario controls	Quebec controls	Combined controls
No. of analyzed subjects∗	663	889	267	1156
No. of *FLG* heterozygotes†	112	94	29	123
No. of *FLG* homozygotes/compound heterozygotes‡	18	4	1	5
Total no. with 1 or 2 *FLG* mutations	130	98	30	128
Proportion with 1 or 2 *FLG* mutations	0.196	0.110	0.112	0.110
OR	NA	1.97	1.93	1.96
95% CI	NA	1.47-2.65	1.24-3.06	1.49-2.58
χ^2^ test (*P* value)	NA	22.33 (2.30 × 10^−6^)	9.37 (2.21 × 10^−3^)	25.22 (5.12 × 10^−7^)

*NA*, Not applicable.

∗ Genotyping failures occurred in 11 PA cases, 5 of the Ontario controls, and 1 of the Quebec controls.

† One *FLG* mutation detected.

‡ Two of the same (*FLG* homozygote) or 2 different (compound heterozygote) *FLG* mutations detected.

**Table III:** PA case definition analysis (PA cases compared with the combined control group)

	No. of subjects	OR	95% CI
Minimum criteria to be considered for inclusion: a. Convincing history of PA∗ **and** (i) SPT ≥3 mm **or** (ii) psIgE ≥ 0.35 kU/L **or** b. No history of peanut ingestion/uncertain history of allergy **and** (i) SPT ≥3 mm **and** (ii) psIgE ≥15 kU/L **or** c. Any history suggestive of an IgE-mediated reaction not compatible with anaphylaxis **and** (i) SPT ≥8 mm **or** (ii) SPT ≥4 mm if <2 y old **or** (iii) psIgE ≥15 kU/L **or** d. Positive OFC	674	1.96	1.49-2.58
psIgE ≥15 kU/L **or** SPT ≥8 mm, **or** positive OFC†	526	1.97	1.47-2.65
psIgE ≥57 kU/L **or** SPT ≥8 mm, **or** positive OFC	486	2.07	1.53-2.78
psIgE ≥57 kU/L **or** SPT ≥15 mm, **or** positive OFC	267	2.07	1.42-2.96
psIgE ≥15 kU/L, **or** SPT ≥8 mm **AND** anaphylaxis, **or** positive OFC	266	2.09	1.44-3.00
psIgE ≥57 kU/L, **or** SPT ≥8 mm **AND** anaphylaxis, **or** positive OFC	253	2.21	1.52-3.18
psIgE ≥57 kU/L, **or** SPT ≥15 mm **AND** anaphylaxis, **or** positive OFC	122	2.28	1.38-3.69

*OFC*, Oral food challenge; *psIgE*, peanut-specific immunoglobulin E; *SPT*, skin prick test.

∗ A convincing history was defined as a minimum of 2 mild symptoms/signs or either 1 moderate or 1 severe symptom/sign occurring within 120 min after peanut contact or ingestion. (1) Mild: pruritus, urticaria, flushing, and/or rhinoconjunctivitis. (2) Moderate: angioedema, throat tightness, change in voice, coughing, difficulty breathing (other than wheeze), nausea and/or vomiting, and/or abdominal pain. (3) Severe: wheezing, stridor, cyanosis, and/or circulatory collapse.

† The definition used in our previous work^1^ required a history of anaphylaxis or a history suggestive of type I hypersensitivity to peanut along with SPT ≥8 mm and psIgE ≥15 kU/L.

The self-reported prevalence of asthma was 11% in the Ontario controls, compared with 65% in the PA cases (see Table E5 in this article's Online Repository at www.jacionline.org). Univariate analysis found PA status to be the strongest predictor of a mutation, followed by asthma. Neither age nor gender had an appreciable relationship with the presence of *FLG* mutations on univariate or multivariate analysis and were not included in the final model. Multivariate logistic regression found no evidence for an effect of asthma (OR, 1.12; 95% CI, 0.79-1.59) on the relationship between PA (OR, 1.81; 95% CI, 1.29-2.55) and *FLG* LOF mutations. The proportion of mutations was similar in those PA cases with and without asthma. Peanut-allergic individuals with asthma had at least 1 mutation in 19.8% of the cases (95% CI, 0.159-0.236), while 18.7% (95% CI, 0.136-0.238) of the PA cases without asthma had at least 1 mutation.

An additional analysis using a constructed atopic asthma variable to control for the effect of smoking yielded similar results. PA status remained significant (OR, 1.88; 95% CI, 1.27-2.80), while history of atopic asthma was not (OR, 1.44; 95% CI, 0.59-3.53). Results were also unchanged after the sensitivity analysis of the self-reported asthma variable, with only PA status remaining significant in the multivariate analysis. The sensitivity analysis on PA status similarly had little effect on the findings. While 20% resolution in cases alone finds that both PA and asthma status are nonsignificant, modeling 20% resolution of cases with 1% prevalence in the general population finds that only PA status remains significant in the multivariate model.

This study substantiates the relationship between PA and *FLG* LOF mutations, and moreover this relationship appears independent of diagnostic criteria of PA and history of asthma, although residual confounding is always possible. All sets of diagnostic criteria used in this study were intended to define individuals with clinical allergy, although it is possible that some individuals who were merely sensitized could have been included in the less stringent definitions of PA. Despite this possibility, the association between the *FLG* LOF mutations and PA does not appear to vary with the diagnostic criteria for PA. Although the point estimates for the OR increase as the PA definition becomes more stringent, the sample size decreases and the CIs overlap; thus, the differences are not statistically significant.

The similarity of the OR in the 2 control groups provides indirect evidence that it is unlikely that there are common *FLG* mutations not yet identified in French-Canadians, under the assumption of similar prevalence of PA. If unidentified common French-Canadian *FLG* variants exist, one would expect the observed OR to be higher when the PA cases were compared with the Quebec controls, due to a lower detection of mutations in the control group. However, further evidence such as the sequencing of *FLG* in French-Canadians would be of interest.

Limitations of this study include the inability to verify ethnicity by using genetic markers of ethnicity, which were not available for either the cases or controls. However, of the 47 identified *FLG* mutations, those examined here are shared in many European populations^2^ (see Table E6 in this article's Online Repository at www.jacionline.org), including the expected ancestries of our case and control groups, giving us confidence that population stratification is not the cause of the difference in mutation frequency in the cases and controls. The rate of mutations in the control groups is comparable to the number seen in other Caucasian general populations (Table E6), which also leads us to believe that we have not overestimated the association. This study was also impeded by the inability to confirm asthma status by diagnostic means and that the only control group for which we had asthma and smoking history was a group of adults. While genotype will not change with age, the age difference could have affected ethnicity (eg, through different times of immigration) and PA status because of generational lifestyle, dietary, or environmental differences. Asthma status could also be affected by this age difference, because of smoking history, failure to remember a history of childhood asthma, as well as the possibility that cases have not yet developed asthma, although there is already a large proportion of PA cases who report asthma. In an effort to address these issues, sensitivity analyses of both PA and smoking status were undertaken, which found no appreciable effects on the findings of this study.

The results of this study lend credence to the hypothesis that sensitization in allergic disease in at least some patients may occur through the skin.^10^ Household peanut consumption is a risk factor for the development of PA^11^ and may be a proxy for the amount of cutaneous peanut exposure for a young child. Sensitization to peanut proteins may be related to cross-sensitization to aeroallergens, such as birch pollen proteins that share homology with peanut allergens.^12^ As filaggrin is not expressed beyond the inferior turbinates, perhaps exposure to these homologous antigens through the skin or mucosa could result in cross-sensitization to peanut. If true, therapies targeted toward the skin, to either improve barrier function or decrease inflammation, may prevent allergic disease. Continued research in barrier function, immunologic mediators, and environmental interactions is needed to further knowledge of the pathogenesis of atopic diseases.

## Figures and Tables

**Table E1:** rs and accession numbers of screened *FLG* mutations

Mutation	Accession number	rs number
R501X	CM061002	rs61816761
2282del4	CD061417	rs41370446
R2447X	CM071765	rs138726443
S3247X	CM071767	rs150597413

**Table E2:** Laboratory and clinical cutoffs used for case definitions

Criterion	Cutoff	References
Peanut-specific IgE	≥0.35 kU/L	^E4^
	≥15 kU/L	E5, E6, E7
	≥26.5 kU/L	^E8^
	≥57 kU/L	^E9^
Skin prick test	≥3 mm	^E10^
	≥8 mm	E5, E6, E7^,^^E11^^,^^E12^
	≥15 mm	^E9^^,^^E13^
Oral food challenge	Positive	^E5^
Anaphylaxis	2 systems involved among mucocutaneous, respiratory, gastrointestinal, and cardiovascular systems	^E14^

**Table E3:** Thirteen case definitions of PA

	Case definition
1.	a. Convincing history of an allergic reaction to peanut **and** (i) SPT of ≥3 mm to peanut **or** (ii) psIgE ≥0.35 kU/L **or** b. No history of peanut ingestion/uncertain history of PA **and** (i) SPT of ≥3 mm **and** (ii) psIgE ≥15 kU/L **or** c. Any history suggestive of an IgE-mediated reaction not compatible with anaphylaxis as defined above **and** (i) SPT of ≥8 mm **or** (ii) SPT of ≥4 mm if <2 y of ageE11, E17 **or** (iii) psIgE ≥15 kU/L **or** d. Positive OFC
2.	psIgE ≥15 kU/L **or** SPT ≥8 mm **or** positive OFC
3.	psIgE ≥26 kU/L **or** SPT ≥8 mm **or** positive OFC
4.	psIgE ≥57 kU/L **or** SPT ≥8 mm **or** positive OFC
5.	psIgE ≥15 kU/L **or** SPT ≥15 mm **or** positive OFC
6.	psIgE ≥26 kU/L **or** SPT ≥15 mm **or** positive OFC
7.	psIgE ≥57 kU/L **or** SPT ≥15 mm **or** positive OFC
8.	psIgE ≥15 kU/L **or** SPT ≥8 mm **and** anaphylaxis, **or** positive OFC
9.	psIgE ≥26 kU/L **or** SPT ≥8 mm **and** anaphylaxis, **or** positive OFC
10.	psIgE ≥57 kU/L **or** SPT ≥8 mm **and** anaphylaxis, **or** positive OFC
11.	psIgE ≥15 kU/L **or** SPT ≥15 mm **and** anaphylaxis, **or** positive OFC
12.	psIgE ≥26 kU/L **or** SPT ≥8 mm **and** anaphylaxis, **or** positive OFC
13.	psIgE ≥57 kU/L **or** SPT ≥8 mm **and** anaphylaxis, **or** positive OFC

*OFC*, Oral food challenge; *psIgE*, peanut-specific immunoglobulin E; *SPT*, skin prick test.

**Table E4:** Number of subjects with *FLG* genotypes in PA cases and controls

Genotype	n (%)		
	PA cases	Ontario controls	Quebec controls
NMC; NMC∗	533 (80.4)	791 (88.9)	237 (88.8)
R501X; NMC	53 (8.0)	32 (3.6)	13 (4.9)
2282del4; NMC	39 (5.9)	44 (4.9)	10 (3.7)
R2447X; NMC	10 (1.5)	10 (1.1)	4 (1.5)
S3247X; NMC	10 (1.5)	8 (0.9)	2 (0.7)
R501X; R501X	3 (0.5)	0	0
2282del4; 2282del4	1 (0.2)	0	1 (0.4)
S3247X; S3247X	0	1 (0.1)	0
R501X; 2282del4	7 (1.1)	1 (0.1)	0
R501X; R2447X	3 (0.5)	0	0
R501X; S3247X	0	1 (0.1)	0
2282del4; R2447X	2 (0.3)	1 (0.1)	0
2282del4; S3247X	2 (0.3)	0	0
Genotype failure	11	5	1
Totals	674	894	268

∗ Nonmutation carrier; ie, none of the 4 *FLG* mutations found.

**Table E5:** Asthma, emphysema, and smoking history: PA cases and controls

	PA cases	Ontario controls
No. of subjects	674	894
Asthma		
No. with asthma	426	98
Proportion with asthma (CI)	0.645 (0.609-0.682)	0.110 (0.090-0.131)
No. with missing data	14	5
Bronchial emphysema		
No. with bronchial emphysema	NA	38
Proportion with bronchial emphysema (CI)	NA	0.043 (0.029-0.056)
No. with missing data	NA	2
Smoking		
No. of smokers	NA	496
Proportion of smokers (CI)	NA	0.557 (0.524-0.589)
No. with missing data	NA	3

*NA*, Not applicable.

**Table E6:** *FLG* mutation frequencies in European populations

Population	Mutation frequency (%)	References
English	8-14	E18, E19
Irish	4-8	E20, E21
German	6	^E22^
Austrian	6	^E23^
Danish	7-8	E24, E25
French	4	E26, E27
Polish	5	^E28^

## Methods

Although not predictive of reaction, the size of the skin prick wheal is correlated to the probability of reaction on food challengeE1, E2 while the level of peanut-specific immunoglobulin E is correlated to anaphylaxis on food challenge.^E3^ After review of the literature, several case definitions were evaluated by using the criteria outlined in Table E2.

Because there is some evidence that the use of multiple test types may improve the specificity of PA diagnosis,E13, E15, E16 various arrangements of these criteria were evaluated by combining each peanut-specific immunoglobulin E level *and* each skin prick test cutoff, or combining each peanut-specific immunoglobulin E level *or* each skin prick test cutoff. These criteria were also combined both with and without a clinical history of anaphylaxis as defined by the consensus statement on anaphylaxis.^E14^ These parameters led to a continuum of 13 separate case definitions for PA (Table E3).
